# First person – Kingsley Ugwuagbo and Sujit Maiti

**DOI:** 10.1242/bio.043349

**Published:** 2019-04-15

## Abstract

First Person is a series of interviews with the first authors of a selection of papers published in Biology Open, helping early-career researchers promote themselves alongside their papers. Kingsley Ugwuagbo and Sujit Maiti are co-first authors on ‘Prostaglandin E2 promotes embryonic vascular development and maturation in zebrafish’, published in BiO. Kingsley is an MSc student in the lab of Dr Mousumi Majumder at Brandon University, Canada, investigating early detection strategies in breast cancer. Sujit is a research assistant in the lab of Dr Mousumi Majumder at Brandon University, investigating the simplest approach to discovering early detection of breast cancer.

**What is your scientific background and the general focus of your lab?**


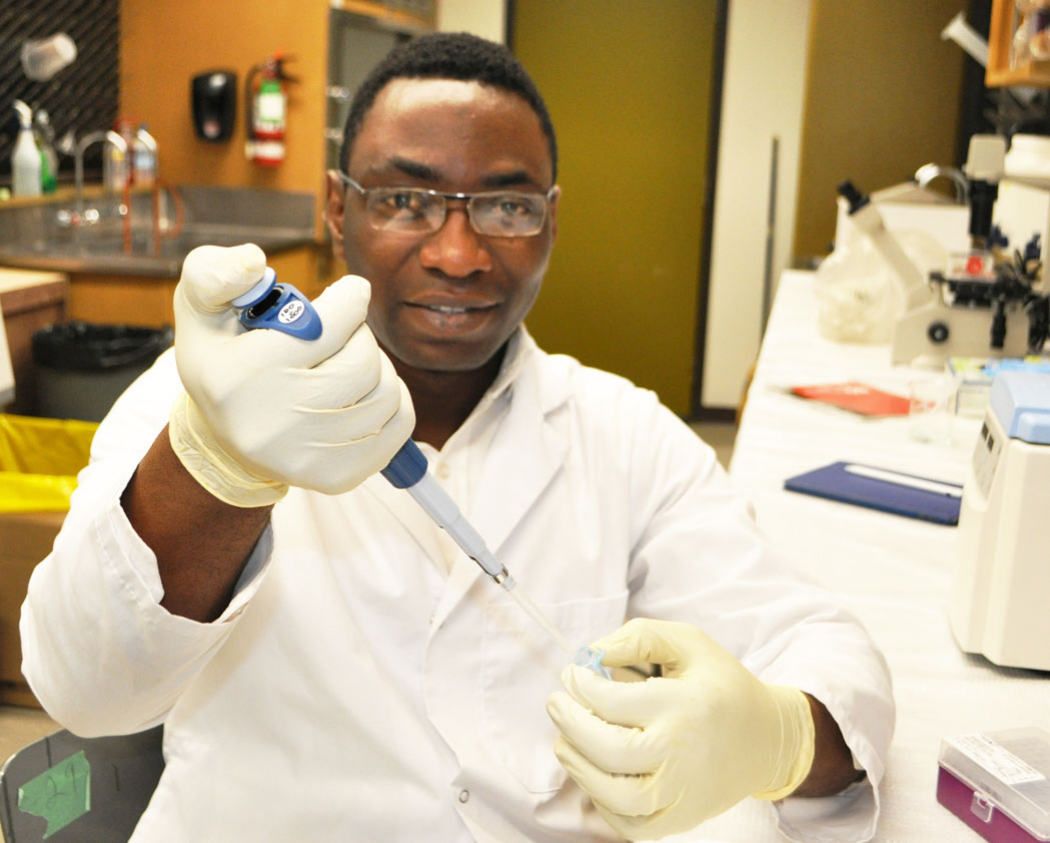


**Kingsley Ugwuagbo**

**KCU:** I was working as a cytopathologist in Nigeria before coming to Canada as an MSc student in 2017. Right now, I am a graduate student at Dr Majumder's laboratory in the department of biology at Brandon University in Canada. Our focus is to investigate the roles of prostaglandin E2 (PGE2) and PGE2-induced small non-coding RNAs (miRNAs) in breast cancer metastasis. I am completing my thesis on establishing the roles of PGE2-induced miRNAs in tumor-associated angiogenesis and lymphangiogenesis in breast cancer.


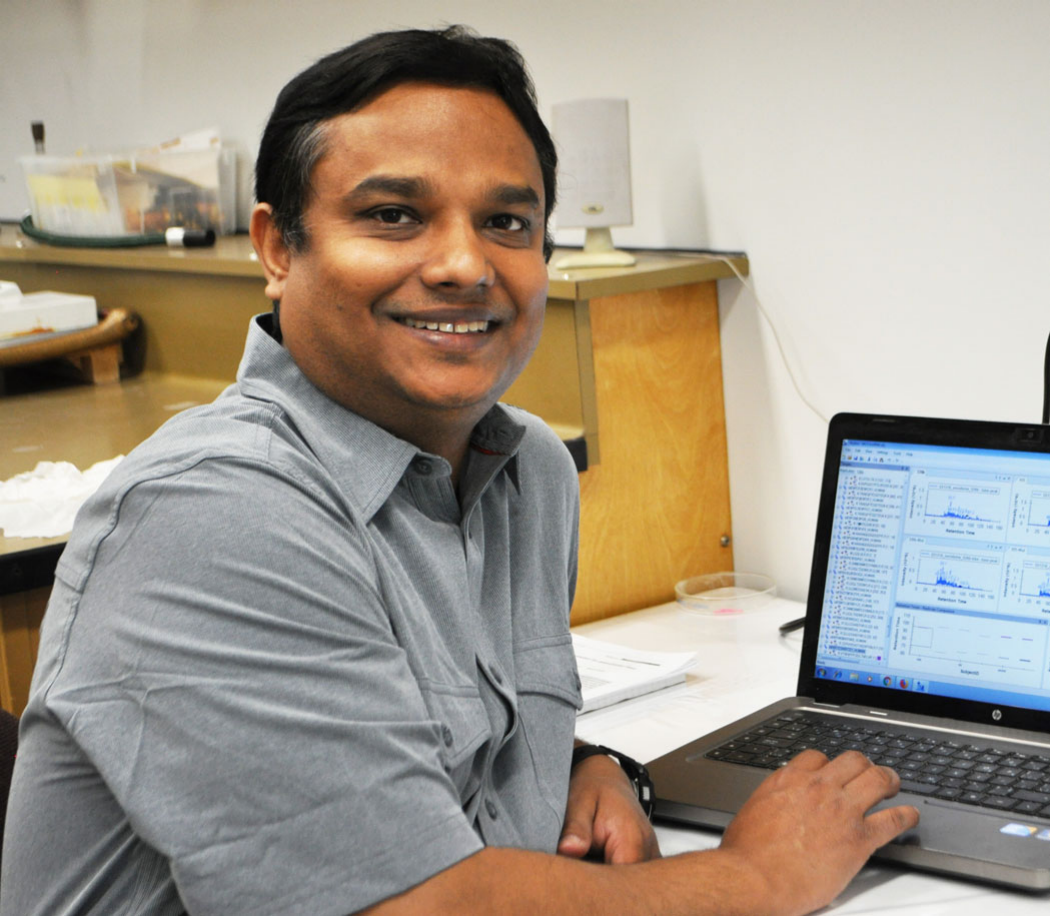


**Sujit Maiti**

**SM:** I am a bioinformatician; I gained experience in analyzing genome, transcriptome and proteome data in clinical research projects to identify variants associated with disease over a decade. I have earned two MSc degrees, one in molecular genetics and one in computer application. With my training in both disciplines, my curiosity is to analyze cell secretome (protein secreted from cells) data from miRNA high cells to identify miRNA secretory proteins. This project could help identify proteins upregulated by miRNA to induce angiogenesis and we shall further investigate cell signaling pathways regulated by miRNAs.

Majumder's laboratory is focused on decoding the roles of miRNA in breast cancer metastasis. The long-term goal of the laboratory is to identify novel breast cancer biomarker for early detection.

**How would you explain the main findings of your paper to non-scientific family and friends?**

PGE2 is produced normally by all vertebrates, including humans, to control embryo growth and development. However, in adults, overproduction of PGE2 causes pain and is associated with human breast cancer progression. Thus, understanding the pathways of different cellular and physiological events mediated by PGE2 is key for anti-cancer drug research. The main finding in this article is that PGE2 regulates vital physiological events involved in vertebrate development by promoting growth and maturation of new blood vessels in zebrafish. With PGE2 injected in embryos, we observed that growth and development of zebrafish was stimulated like early hatching, with improved heartbeat and maturation of newly developed blood vessels. This study shows the novel roles of PGE2 in vertebrates and the results can be translated to human to make a cautionary note on the use of pain medications which block PGE2 production.

**What are the potential implications of these results for your field of research?**

A part of our investigation focuses on the roles of PGE2 in microRNA regulation and anti-inflammatory drugs (PGE2 inhibitors) on microRNA expression in breast cancer. We found that microRNAs are involved in making new blood vessels used to spread breast cancer cells to other organs of the body and could be used as blood biomarkers for early detection of breast cancer. Interestingly, the findings in this paper show that PGE2 mediates important cellular events involved in vertebrate development and maturation of new blood vessels. Hence, use of anti-inflammatory drugs (PGE2 blockers) could easily stop normal cell functions mediated by PGE2. This identifies PGE2 as a key regulator in vertebrate development and functions. Thus, our research places a cautionary note on the use of NSAIDs. It also establishes zebrafish as a good *in vivo* model for anti-cancer drug researches.

**Figure:**
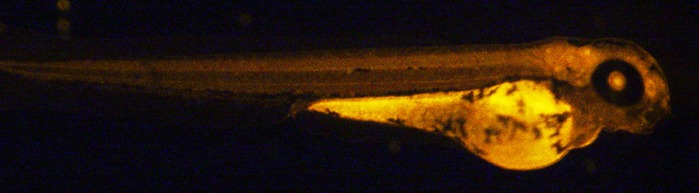
**Hatching process of a normal zebrafish embryo.**

**What has surprised you the most while conducting your research?**

In our experiment, we microinjected only tiny amounts of PGE2 into fertilized zebrafish eggs and observed the growth of the embryo into the larval forms. It was amazing to see that new blood vessel development is significantly faster in PGE2-injected embryos than non-injected ones.

**What, in your opinion, are some of the greatest achievements in your field and how has this influenced your research?**

While the last decade has been about generating human whole genome data, this era is all about finding the importance of non-coding RNA to understand diseases. Ground-breaking research is going on to understand the roles of miRNA in cancer and that's what influenced this research.

“Ground-breaking research is going on to understand the roles of miRNA in cancer and that's what influenced this research.”

**What changes do you think could improve the professional lives of early-career scientists?**

For us, doing research is a passion and not a routine job. The passion to learn new things in science for the good of mankind remains paramount; not minding that we might not earn enough money to run our families smoothly. The insufficient income as a graduate student or research assistant and high tuition payments limit many young scientists from attaining big feats in research. More funding for the training of young researchers would go a long way to inspire them to accomplish their professional goals.

**What's next for you?**

**KCU:** I have spent over 10 years on routine breast cancer tissue analysis at the clinic. Presently, I am learning cutting-edge molecular biological approaches to detect breast cancer at an early stage. My purpose is to bridge the gap that currently exists between the research bench and the bedside. I wish to develop a more stable and less invasive biomarker for early detection of breast cancer.

**SM:** I left my IT career to pursue my passion for biological research as a bioinformatician. High throughput data generation is now affordable, but the problem is analysis and identification of variants with biological significance. Analyzing data carefully and correctly helps to identify unknown markers in different complex human diseases like cancer. Maybe one day I can help to uncover the secret behind it. I will continue my career as a biological data analyst to help improve human quality of life.
